# Comparison of methylation estimates obtained via MinION nanopore sequencing and sanger bisulfite sequencing in the *TRPA1* promoter region

**DOI:** 10.1186/s12920-023-01694-6

**Published:** 2023-10-23

**Authors:** Sara Gombert, Kirsten Jahn, Hansi Pathak, Alexandra Burkert, Gunnar Schmidt, Lutz Wiehlmann, Colin Davenport, Björn Brändl, Franz-Josef Müller, Andreas Leffler, Maximilian Deest, Helge Frieling

**Affiliations:** 1 Laboratory for Molecular Neuroscience, Department of Psychiatry, Social Psychiatry and Psychotherapy, Hannover Medical School, Hannover, Germany; 2 Department of Human Genetics, Hannover Medical School, Hannover, Germany; 3 Research Core Unit Genomics, Hannover Medical School, Hannover, Germany; 4 Department of Genome Regulation, Max Planck Institute for Molecular Genetics, Berlin, Germany; 5 Department of Psychiatry and Psychotherapy, Zentrum für Integrative Psychiatrie gGmbH, University Medical Center Schleswig-Holstein, Campus Kiel, Kiel, Germany; 6 Department of Anesthesiology and Intensive Care Medicine, Hannover Medical School, Hannover, Germany

**Keywords:** TRPA1, Epigenetics, Methylation, Bisulfite sequencing, Nanopore sequencing, Cas9-mediated PCR-free enrichment, guideRNAs

## Abstract

**Background:**

Bisulfite sequencing has long been considered the gold standard for measuring DNA methylation at single CpG resolution. However, in recent years several new approaches like nanopore sequencing have been developed due to hints for a partial error-proneness of bisulfite sequencing. Since these errors were shown to be sequence-specific, we aimed to verify the methylation data of a particular region of the *TRPA1* promoter from our previous studies obtained by bisulfite sequencing.

**Methods:**

We compared methylation rates determined by direct bisulfite sequencing and nanopore sequencing following Cas9-mediated PCR-free enrichment.

**Results:**

We could show that CpG methylation levels above 20% corroborate well with our previous data. Within the range between 0 and 20% methylation, however, Sanger sequencing data have to be interpreted cautiously, at least in the investigated region of interest (*TRPA1* promotor region).

**Conclusion:**

Based on the investigation of the TRPA1- region as an example, the present work can help in choosing the right method out of the two current main approaches for methylation analysis for different individual settings regarding many factors like cohort size, costs and prerequisites that should be fulfilled for each method. All in all, both methods have their raison d’être. Furthermore, the present paper contains and illustrates some important basic information and explanation of how guide RNAs should be located for an optimal outcome in Cas9 mediated PCR free target enrichment.

**Supplementary Information:**

The online version contains supplementary material available at 10.1186/s12920-023-01694-6.

## Introduction

Bisulfite sequencing, developed by Frommer and colleagues [1], is based on the sodium bisulfite-mediated conversion of cytosine to uracil in single-stranded DNA, and has long been considered the gold standard for methylation analysis. However, this method is susceptible to errors due to the bisulfite conversion and the subsequent amplification of DNA strands, which can lead to misinterpretation of the results. The harsh chemical treatment of DNA leads to significant degradation, thereby causing bisulfite conversion errors [2, 3]. Therefore, a balanced control between the desired conversion of unmethylated cytosines to uracils and the undesired DNA degradation and inappropriate conversion of methylated cytosines to thymines is indispensable. Otherwise, the unpredictable level of false positive and false negative results may be elevated due to the differing conversion efficiencies of cytosines depending on the sequence context [2]. However, conversion errors are relatively low when using modern kits for bisulfite treatment [4, 5], whereas recovery rates of available DNA for sequencing are between 18 and 50% [5]. The amplification of the target regions in bisulfite sequencing imposes the risk of intensifying biases, primarily due to the elevated error rates in high- and low-GC regions [6]. The amplification of artefacts from sequence-specific bisulfite-induced degradation and conversion errors leads to a higher overall bias in protocols involving amplification [7]. Since some regions are more susceptible to biases than others [7], the error-proneness of a sequence of interest is hard to predict. The choice of bisulfite conversion protocol or polymerase significantly reduces these artefacts but cannot completely abolish them [7]. In contrast, nanopore sequencing of native DNA measures cytosine methylation directly and does not require error-prone procedures such as bisulfite treatment or amplification of target regions. Nanopore sequencing is able to discriminate between the four standard bases by measuring the change in current as DNA or RNA molecule translocates through a protein nanopore. Furthermore, a methylated cytosine also differs from an unmethylated cytosine by a measurable change in current, thus enabling a real-time methylation sequencing without any prior labeling or modification [8–12].

To evaluate the reliability of our previous *TRPA1* promoter methylation studies [13, 14], we compared the methylation rates obtained via bisulfite and nanopore sequencing. For this purpose, we used the Cas9-mediated PCR-free enrichment to target the *TRPA1* promoter region for subsequent MinION nanopore sequencing. This targeted sequencing approach enables the enrichment of loci of interest, yielding high coverage of the desired genomic regions [15], which is necessary to enable a reliable evaluation of methylation rates. The absolute minimum number of reads required might depend on the target region and the methylation level.

## Materials and methods

### Samples

Blood for comparison of methylation rates obtained via Sanger bisulfite and nanopore sequencing in 10-plicates and 12-plicates, respectively, was drawn from a healthy volunteer recruited in Hannover before DNA extraction from buffy coat by the Hannover Unified Biobank. Approval for analysis of the healthy control was obtained at the Ethics Committee of the Hannover Medical School (Permit Number 2842 − 2015).

For comparison with Sanger bisulfite sequencing- results of healthy subjects from a previous study [13], DNA extraction from whole blood was performed as stated below (n = 10) in order to obtain DNA of high quality and yield for nanopore sequencing.

For comparison with Sanger bisulfite sequencing- results of Crohn patients from another previous study [14], the DNA that was already extracted from whole blood as stated in the published manuscript was used (n = 5), due to the unavailability of fresh blood samples.

### DNA extraction

800 µl of whole blood were incubated with 80 µl Proteinase K (Macherey-Nagel, Düren, Germany) for 15 min at room temperature and centrifuged at 21 000 x g and 4 °C for 5 min. The resulting 200 µl-fractions were separated, and the Nucleo-Mag Blood 200 µl DNA Kit (Macherey-Nagel, Düren, Germany) was used to extract and clean-up genomic DNA. For pipetting and transferring steps, and for the purification of DNA, a Biomek N x P (Beckman Coulter, Brea, CA) was used. DNA concentration was determined on a DeNovix DS-11 Spectrophotometer (DeNovix, Wilmington, USA), and the fraction with the highest DNA concentration (about 200 ng/µl) of each sample was used for nanopore sequencing after confirmation of high-molecular weight of the extracted DNA by pulsed-field gelelectrophoresis (Blue Pippin®).

### Sanger bisulfite sequencing

DNA samples were bisulfite converted and purified using the EpiTect 96 Bisulfite Kit (QIAGEN, Hilden, Germany). Amplification of the *TRPA1* promoter target sequence on chromosome 8 using the forward primer 5’-GTTTGTATTAGATAGTTTTTTTGTTTG-3’ (position − 819 to -792 in relation to the first base in exon 1) and the reverse primer 5’-TCCTACAAACCTATATTTCCCAC-3’ (position − 441 to -418) (product length 401 bp), purification of the amplified target sequence, and sequencing on a 3500xL Genetic Analyzer (ABI Life Technologies, Carlsbad, USA) was performed as described previously [13]. All samples showed a quality value above 20 for trace score in the Sequence Scanner Software (ABI Life Technologies, Carlsbad, USA). Methylation rates for each CpG site were determined via the Epigenetic Sequencing Methylation Analysis Software [16].

In order to enable comparison with our previous *TRPA1-*studies we retained the nomenclature of all investigated CpGs (for example − 412), which is the position of the respective CpG in relation to the first base in exon 1. However, it has to be mentioned that in the meantime, the attribution of the starting point of exon 1 in the genomic sequence has been changed. While exon 1 has originally been considered to start at GRCh38 position 72.075.617, the first base of exon 1 is now allocated at GRCh38 position 72.075.584, resulting in a difference of 33 bp. Therefore, if one is interested in the current relative position of the investigated CpGs from the first base of exon 1, one has to subtract 33 from the old position (for example: -412–33 = -445). Corresponding GRCh38 positions for all CpGs are given in supplemental table S1. Relative primer positions in the text above are already given in relation to the current starting position of *TRPA1* exon 1 at 72.075.584.

### Nanopore sequencing

Guide RNAs (Table 1) were designed using the target prediction program CHOPCHOP (http://chopchop.cbu.uib.no) and were ordered from IDT (Integrated DNA Technologies, Coralville, USA).

**Table 1:** Guide RNAs used for establishing the Cas-mediated PCR-free enrichment of the *TRPA1* promoter sequence analyzed via nanopore sequencing. Different combinations were tested, with a combination of guide RNA # 4 and # 9 giving the highest number of calls per site. Guide RNAs # 1, # 3 and # 8 have to be assumed to be “interfering”

Guide RNA #	Sequence	PAM	GRCh38 genomic position
1	TTGCCACAAAGAGATCAAGT	AGG	chr.8: 72,070,598 to 72,070,579
2	GAGTATGGTACACCTTCTTG	AGG	chr.8: 72,071,344 to 72,071,363
3	GCACACAACAGAAATGTAGA	AGG	chr.8: 72,070,845 to 72,070,826
4	AGCAATTTTGTGATCCCCTA	AGG	chr.8: 72,070,732 to 72,070,751
5	GAACAAAGACACTCGCTCAA	TGG	chr.8: 72,081,680 to 72,081,661
6	TGGCATGTTAAGACAATTGT	TGG	chr.8: 72,082,102 to 72,082,083
7	GAGCTTCTAATCAGTGACTG	AGG	chr.8: 72,080,169 to 72,080,150
8	ACTTATGCTTACCATTCAGA	TGG	chr.8: 72,080,463 to 72,080,444
9	CCAGTAACATATGAAAAGGT	TGG	chr.8: 72,080,463 to 72,080,444

PAM: Protospacer Adjacent Motif

For the selection of guide RNAs the following quality criteria were applied: efficiency ≥ 0.5, self-complementarity max. 2, GC content 40–70%, mismatches between off-targets and guide RNA: MM0 (no mismatch) = 0, MM1 (1 mismatch) = 0, MM2 (2 mismatches) as low as possible, MM3 (3 mismatches) as low as possible. Several guide RNAs per cutting site were identified and subsequently, their quality was assessed using the online tool Off-spotter (https://cm.jefferson.edu/Off-Spotter). Guide RNAs with off-target mismatches close to the PAM (protospacer adjacent motif) were preferred over those with off-target mismatches far from the PAM due to a reduced binding and cutting probability of the Cas9 enzyme. The DNA quality was assessed via pulsed-field gel analysis using a Pippin Pulse electrophoresis power supply (Sage Science, Beverly, USA). 5 µg of high-molecular weight DNA of each sample were used for the Cas-mediated PCR-free enrichment using the Ligation Sequencing (SQK-LSK109) Kit (Oxford Nanopore Technologies, Oxford, UK) and the Native Barcoding Expansion 1–12 (PCR-free) (EXP-NBD104) Kit (Oxford Nanopore Technologies, Oxford, UK) in the following order: Dephosphorylating genomic DNA, Preparing the Cas9 ribonucleoprotein complexes (RNPs), Cleaving and dA-tailing target DNA, Native barcode ligation with consecutive AMPure XP bead purification, Adapter ligation (during this step buffer AMII instead of buffer AMX and no nuclease-free water was added to the ligation mixture due to prior barcoding), AMPure XP bead purification (TE buffer and AMPure XP beads were scaled up due to the higher final volume after barcoding maintaining the recommended TE to ligation mix volume ratio (1x) and afterwards sample to beads volume ratio (0.3); the pellet was resuspended in 14 µl of preheated elution buffer at 37°C for 20 minutes with flicking the tube every 5 minutes), Priming and loading the SpotON flow cell FLO-MIN106D (Oxford Nanopore Technologies, Oxford, UK), and starting the sequencing run on the MinION (Oxford Nanopore Technologies, Oxford, UK). Raw sequencing data was base-called using guppy v. 2.7 (Oxford Nanopore Technologies, Oxford, UK) with standard settings and config file ”dna_r9.4.1_450bps_hac”. Base-called reads were aligned to GRCh38 using minimap2 [17]. Methylation calling and determination of methylation frequency was performed using nanopolish v 0.12.4 [11].

### Statistical analyses

For statistical calculations and data illustration Prism 5 (GraphPad) was used. For correlation analysis of methylation data between bisulfite and nanopore sequencing, and between CpG − 628 methylation and pressure pain threshold, a *p-value* of ≤ 0.05 was considered significant.

## Results

### Comparison of the bisulfite and nanopore sequencing methods for methylation calling by repeated measurements in one DNA sample

**Fig. 1:**
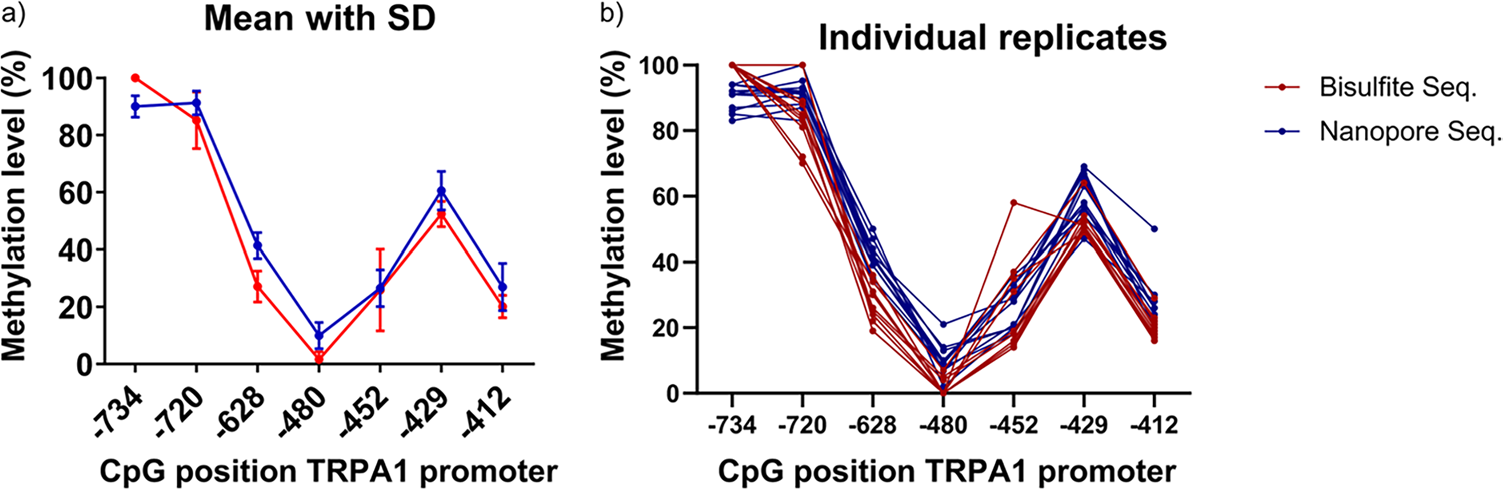
Comparison of methylation rates obtained via direct bisulfite and nanopore sequencing of the TRPA1 promoter. Sanger bisulfite sequencing was performed in 10-plicates, nanopore sequencing in 12-plicates. (a) Mean methylation rates and standard deviation per CpG position, (b) Methylation rates per CpG position of single measurements

Comparing bisulfite and nanopore sequencing, we found similar methylation rates and patterns for the seven CpGs of the *TRPA1* promoter region analyzed in repeated measurements (10 for Sanger bisulfite sequencing, 12 for nanopore sequencing) of DNA extracted from a buffy coat of a healthy volunteer as shown in Fig. 1.

Despite the identical methylation pattern in both methods, it is striking in our graph, that lower absolute values were measured with bisulphite sequencing compared to nanopore sequencing in general. Differences were most evident at CpG positions − 628, -480, -429, and were also visible at CpG − 720 and − 412 (Fig. 1). The only CpG site with higher levels for Sanger bisulfite sequencing compared to nanopore sequencing is CpG − 734. The calculated standard deviation of our measurements is below 10 except at CpG − 452 (Table 2), which mainly resulted from one outlier of the bisulfite sequencing 10-plicates at this CpG position (Fig. 1b). At CpG position of main interest (CpG − 628) the standard deviation is low for both methods (Table 2; Fig. 1a).

**Table 2:** Mean methylation rates and standard deviation of Sanger bisulfite and nanopore sequencing

CpG position	Methylation rate (%)			
	Bisulfite sequencing (10-plicates)		Nanopore sequencing (12-plicates)	
	Mean	SD	Mean	SD
-734	100.0	0.0	90.0	3.8
-720	85.2	9.9	91.3	4.2
-628	27.1	5.4	41.4	4.5
-480	1.6	2.7	10.0	4.6
-452	25.9	14.3	26.6	6.4
-429	52.4	4.5	60.6	6.7
-412	20.1	4.0	26.9	8.3

### Comparison of methylation data obtained via nanopore sequencing with previously published bisulfite sequencing results

**Fig. 2:**
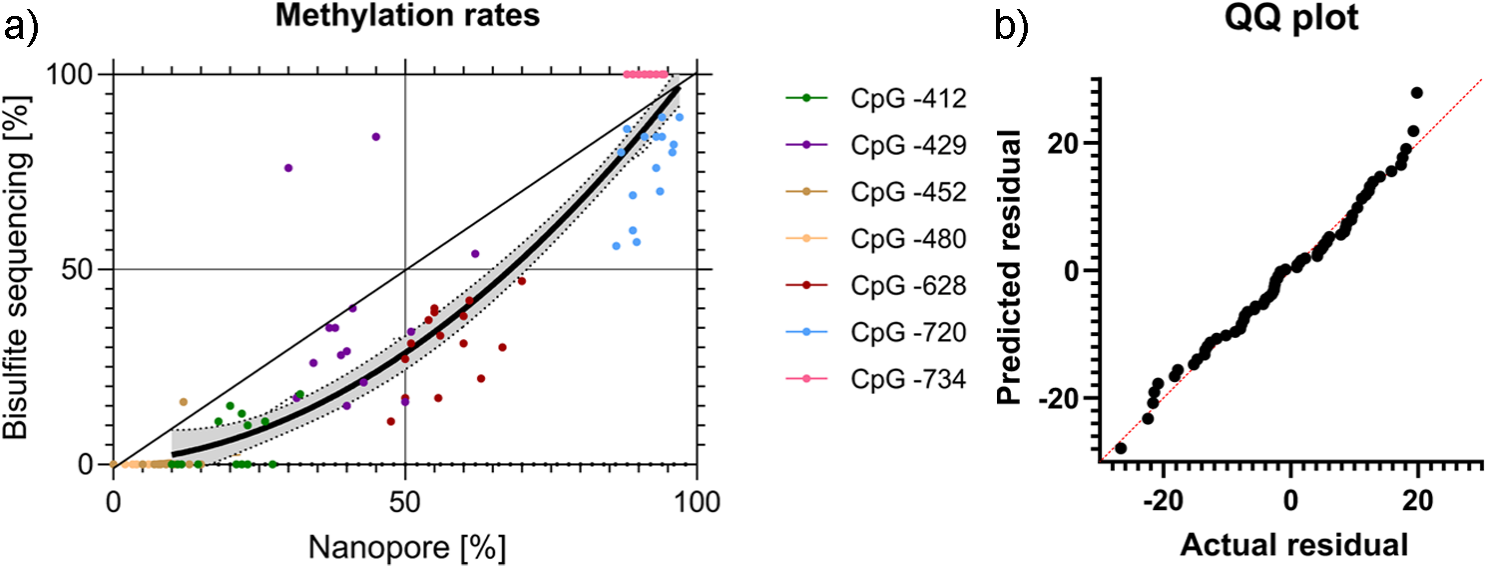
Comparison of methylation data of the *TRPA1* promoter obtained via bisulfite and nanopore sequencing. Sanger bisulfite sequencing results were published previously [13, 14], nanopore sequencing using the same DNA samples was conducted in the present study (from healthy controls and Morbus Crohn patients). A: The relationship between data points generated by nanopore and Sanger sequencing is best described by a non-linear curved fit, showing most prominent mismatches in the methylation range between 0 and 20%; B: A Quantile-Quantile (QQ) plot illustrates the fit of the non-linear regression line

In order to validate our previously published Sanger bisulfite sequencing data of the *TRPA1* promoter [13, 14], we measured the methylation rates via nanopore sequencing in some previously analyzed samples of the same cohorts. Whereas it was possible to re-extract DNA from whole blood samples of healthy controls [13], only DNA extracted previously was available of Crohn patients [14]. Five of these Crohn patient DNA samples possessed a sufficient DNA concentration for nanopore sequencing. The DNA quality was lower than of the DNA extracted from buffy coat or extracted freshly from whole blood, as indicated by pulsed-field gelelectrophoresis showing a higher level of fragmentation. The lower DNA-integrity of samples from the Crohn patient group (n = 5) gave rise to a relatively low number of calls per site of the seven CpGs analyzed, ranging from 16 to 37. The number of calls per site at CpG − 628, our CpG of special interest regarding our previous study varied between 20 and 30. DNA of the healthy control group (n = 10) gave rise to the number of calls per site between 60 and 223 for the seven CpGs, and between 70 and 199 for CpG − 628. Although the experimental conditions were therefore not ideal, the methylation rates measured with the two different methods were relatively congruent (Fig. 2), even though the observation of generally lower methylation rates obtained via Sanger bisulfite sequencing compared to nanopore sequencing could be confirmed (compare Fig. 1). In the methylation range below 20%, this deviation becomes more critical as the values can no longer be distinguished from each other in case of our target region, mainly concerning CpG-480. However, the normal distribution of the residuals was verified by a QQ plot, which illustrates the fit of the non-linear regression line.

**Fig. 3:**
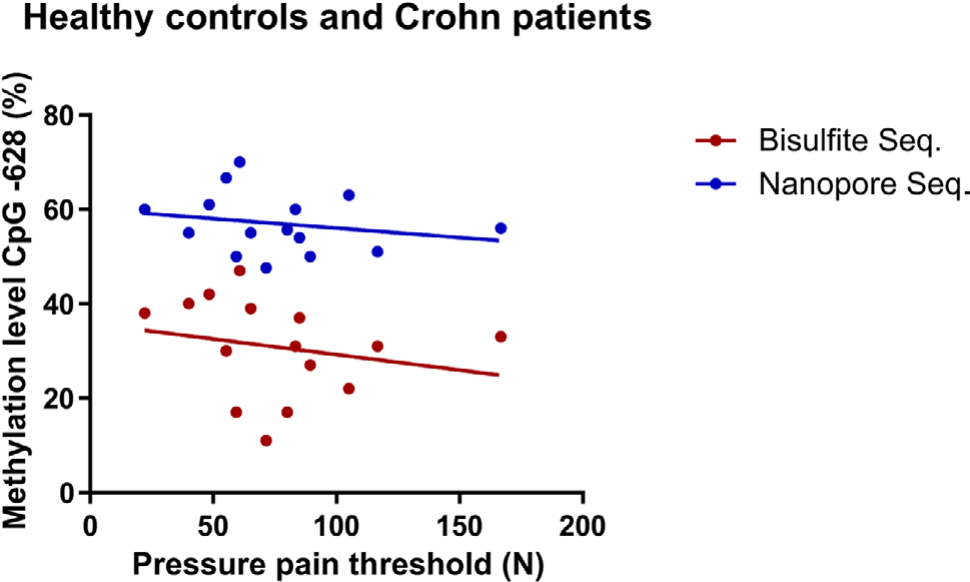
Correlation between CpG − 628 methylation level, determined via bisulfite and nanopore sequencing, and pressure pain threshold. Sanger bisulfite sequencing results and pressure pain thresholds were published previously [13, 14], nanopore sequencing was conducted in the present study. Bisulfite sequencing: R^2^ = 0.049, p = 0.426; nanopore sequencing: R^2^ = 0.048, p = 0.435

In our previous study we found a significant correlation between the methylation of CpG − 628 and pressure pain threshold, measured by an algometer over the thenar muscles [13, 14]. As shown in Fig. 3, the correlation is more pronounced for Sanger bisulfite sequencing data obtained from our previous studies [13, 14] than for nanopore sequencing. For both methods, the correlation did not reach the significance level in the present study with its strongly reduced n-number of participants (due to missing DNA material for Nanopore sequencing), in contrast to the results from our previous studies [13, 14].

### Accuracy of methylation data determined via nanopore sequencing in relation to the number of calls per site

**Fig. 4:**
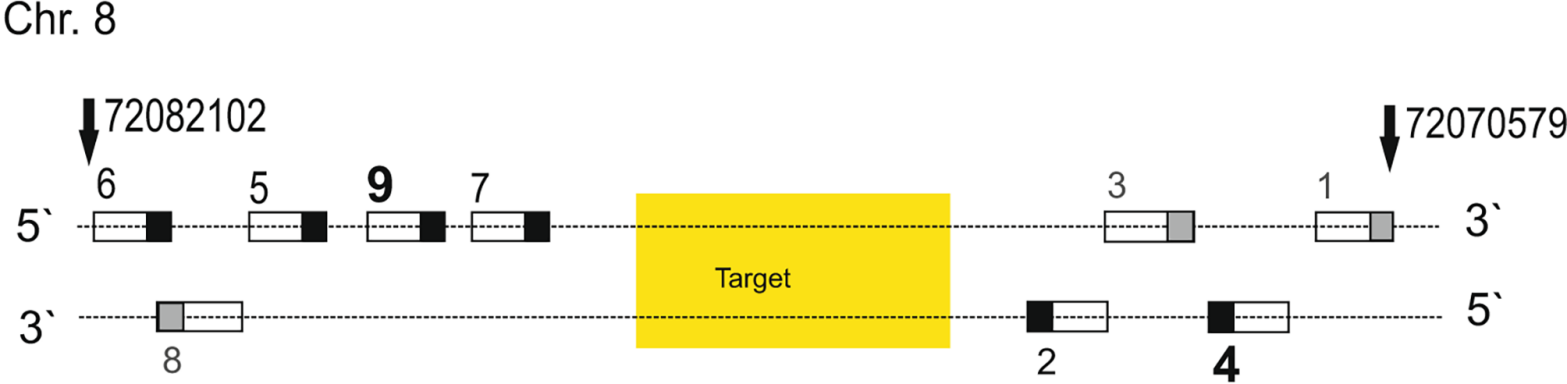
Positions of guide RNAs designed for the target, here the *TRPA1* promoter. Positions of guide RNAs are depicted relative to the target region on chromosome 8. Arrows mark outmost GRCh38 positions. The guide RNAs are numbered (compare Tables 1 and 3) and are depicted as boxes; PAM motifs are indicated by filled squares. The black coloring indicates guide RNAs with a PAM motif pointing towards the target, whereas guideRNAs with a PAM motif facing away from the target (and therefore interfering/ disturbing) are colored in grey. The guide RNAs yielding the highest number of calls per site (# 4 and # 9) are printed in bold

Whereas for DNA sequencing irrespective of base modification calling, obtaining a high number of calls per site is of less importance, a sufficient number is necessary to assess the accurate percentage values of methylation in a mixture of DNA strands. The combination of guide RNA # 4 and # 9 was shown to result in the highest number of calls per site obtained (Table 3). An overview of the relative positions of tested guide RNAs binding to the target region within the *TRPA1* promoter is shown in Fig. 4.

**Table 3:** Calls per site and methylation rates obtained by application of two different guide RNA strategies. Data obtained by use of guide RNAs # 4 and # 9 are based on four experiments (number of calls per site 55–343), data obtained by usage of all guide RNAs are based on five experiments (number of calls per site 17–51). The same DNA sample extracted from a buffy coat was used for all sequencing experiments

Guide RNAs # 4 and # 9							
CpG position		Calls per site				Methylation rate (%)	
bp relative to exon 1	GRCh38 position	Min	Max	Mean	SD	Mean	SD
-734	72,076,349	78	343	195	110	91	0
-720	72,076,335	83	328	182	105	92	2
-628	72,076,243	78	273	164	81	39	3
-480	72,076,095	72	313	176	101	09	1
-452	72,076,067	58	243	139	77	32	3
-429	72,076,044	69	252	140	79	60	5
-412	72,076,027	55	281	154	94	25	4
**All guide RNAs (# 1 - # 9)**							
**CpG position**		**Calls per site**				**Methylation rate (%)**	
**bp relative to exon 1**	**GRCh38 position**	**Min**	**Max**	**Mean**	**SD**	**Mean**	**SD**
-734	72,076,349	26	51	39	10	88	5
-720	72,076,335	23	47	35	10	90	7
-628	72,076,243	18	46	32	11	40	4
-480	72,076,095	20	47	34	10	13	5
-452	72,076,067	18	40	29	7	23	5
-429	72,076,044	19	38	30	9	65	5
-412	72,076,027	17	41	32	12	28	13

In addition to the guide RNA strategy, the number of calls per site highly depends on the concentration and the quality of the DNA used for nanopore sequencing. Therefore, as mentioned before, the number of calls per site obtained when measuring the remaining DNA from Crohn patients extracted for our previous study [14], was relatively low. However, during the establishment of the most suitable sgRNA strategy for our region of interest, we first combined all of the guide RNAs designed (Fig. 4 and Table 3), thereby generating a lower number of reads as compared to the sole application of guide RNAs # 4 and # 9, using the same DNA control sample extracted from buffy coat.

Despite the lower numbers of calls per site obtained by utilizing all guide RNAs, methylation rates were measured within the same range for both guide RNA strategies (Table 3).

**Fig. 5:**
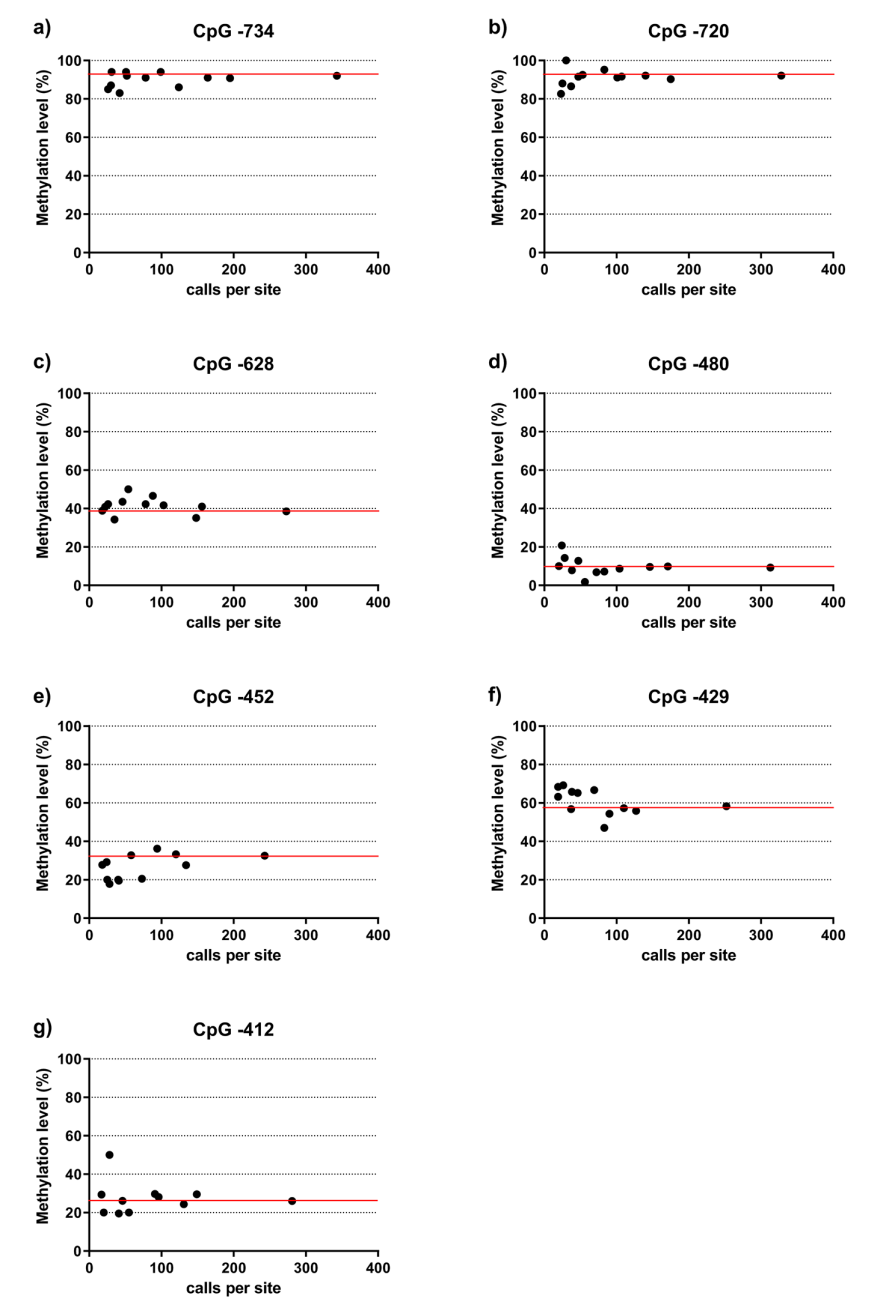
Methylation rates of the *TRPA1* promoter determined via nanopore sequencing in relation to the number of calls per site. Data based on 12 measurements of the same DNA sample. The red line marks the methylation rate measured with the highest number of calls per site per CpG, which is presumably the most accurate value. Several flow cells and different guide RNA strategies for Cas-mediated PCR-free enrichment were used leading to varying numbers of calls per site

Nevertheless, the representation of data points in Fig. 5 reveals that the measurement of methylation rates is not completely stable before reaching a number of calls per site of 100 (or a bit less) for all of the CpGs analyzed. However, deviations are not high, even in the case of low read numbers.

## Discussion

### Repeated measurements in one DNA sample revealed congruent results for methylation calling between the methods Sanger bisulfite and nanopore sequencing

Mean methylation rates and the methylation rates of individual replicates were congruent between bisulfite and nanopore sequencing (Fig. 1), although the number of calls per site varied in nanopore sequencing. The latter will be referred to in the last section (“Biochemical parameters influencing the accuracy of methylation data determined via nanopore sequencing in relation to the number of calls per site”).

Thus, we were able to validate the Sanger bisulfite sequencing results of this particular region of the *TRPA1* promoter. Although various new methods for methylation analysis are available, bisulfite sequencing remains a satisfactory and reliable method with single CpG resolution. A study, which compared the performance of widely used methods for DNA methylation analysis that are compatible with routine clinical use in 18 laboratories in seven different countries, found clonal bisulfite sequencing to perform reasonably well [18]. However, in that comparative study, bisulfite sequencing did not reach the accuracy and reproducibility of the top-ranking methods. The compared methods included bisulfite conversion, PCR amplification, mass spectrometric quantification, microarray analysis, qPCR with a methylation-specific probe, high resolution melting analysis, high-performance liquid chromatography, enzyme-linked-immunosorbent assay, and cloning. The authors mention that until recently, the method of clonal bisulfite sequencing was considered the gold standard for locus-specific DNA methylation mapping, but suggest using one of the novel, less labor-intensive assays for biomarker development. The authors came to the conclusion that amplicon bisulfite sequencing and bisulfite pyrosequencing showed the best all-round performance in their study [18]. Direct bisulfite sequencing is much less labor-intensive than involving plasmid cloning, but, unfortunately, is accompanied by a decrease in accuracy. The extent of this decrease mainly depends on the tools used to calculate the proportion of peak heights between cytosine and thymine. In general, in our study, lower levels were measured with bisulfite sequencing compared to nanopore sequencing (Fig. 1; Table 2). The observation that cytosine modifications seem to have a protective effect against bisulfite treatment-induced DNA degradation, and that bisulfite treatment leads to a depletion of genomic regions enriched for unmethylated cytosines [7], did therefore not concur with our results. A possible explanation for our observation could be a biased amplification of the bisulfite-converted (unmethylated) DNA, which is sequence-dependent and often strand-specific [19]. Since the GC-content of methylated DNA after bisulfite treatment is higher than that of unmethylated DNA, a methodological bias and an inaccurate estimate of methylation in highly methylated regions is possible. The higher melting temperature of the DNA with higher GC-content may increase the likelihood of secondary structure formation for some sequences, and therefore, decrease the PCR efficiency compared to unmethylated sequences 17.

An additional explanation for the lower methylation rates observed with bisulfite sequencing compared to nanopore sequencing could be an inappropriate bisulfite conversion. False-negative data occur with longer incubation times leading to higher degradation and accumulation of inappropriate conversion without necessarily contributing to overall conversion efficiencies [2, 4].

For the sake of completeness, it has to be mentioned, that a detection error in Nanopore sequencing could also account for the difference, even though this is less probable. In general the accuracy of nucleobase identification is 99.99% (Q45) for the pore version R9.4.1 that we used in our nanopore measurements. Nevertheless, accuracy of cytosine methylation estimation depends partly on the type of methylation caller, on the type of genomic region and also on the arrangement of CpGs (singletons vs. non-singletons, the latter referring to CpGs in close proximity to each other) and so on. However, for our data analysis we mainly used nanopolish, which showed a high reliability in different types of genomic regions in a survey and human epigenome wide evaluation study [20].

### Nanopore sequencing confirmed our previously published Sanger bisulfite sequencing results

Methylation rates obtained via nanopore sequencing were quite similar to our previously published bisulfite sequencing results (Fig. 2). As discussed above, the reason why Sanger sequencing detects lower values might be a bias in amplification of bisulfite-treated DNA in advantage of the unmethylated strands. According to the findings obtained from the test DNA (Fig. 1 and results-section “Comparison of the bisulfite and nanopore sequencing methods for methylation calling by repeated measurements in one DNA sample”), the only CpG site with higher levels for bisulfite sequencing compared to nanopore sequencing is CpG − 734, which might be due to the common inaccurate reading at the beginning of the sequence using Sanger sequencing. During the quality control of methylation data carried out in our previous studies, CpG sites with less than 5% inter-individual variability, which applied to CpGs − 734 [13] and − 480 [13, 14], were rejected. However, the methylation rates of all subjects for CpG − 734 were measured around 100%, and those for CpG − 480 around 0% (data not shown). Our direct Sanger bisulfite sequencing approach was not applied for diagnostic purposes of individuals but to gain an overview of the methylation rates of single CpG sites in relation to the subjects’ pain sensitivities within a large cohort [13, 14]. Since a possible bias would apply to all samples independent of the subjects’ pain sensitivity, minor discrepancies are acceptable.

Although correlation of CpG − 628 methylation and pressure pain threshold did not reach significance, which is mainly due to the limited availability of samples with a sufficient quality for Nanopore sequencing (10 healthy subjects, 5 Crohn patients), a trend for low pressure pain thresholds (high pain sensitivities) with high methylation rates at CpG − 628 is still visible (Fig. 3). Remarkably, the pattern are rather similar between Sanger and nanopore sequencing, again revealing a good comparability between both methods.

### Biochemical parameters influencing the accuracy of methylation data determined via nanopore sequencing

**Fig. 6:**
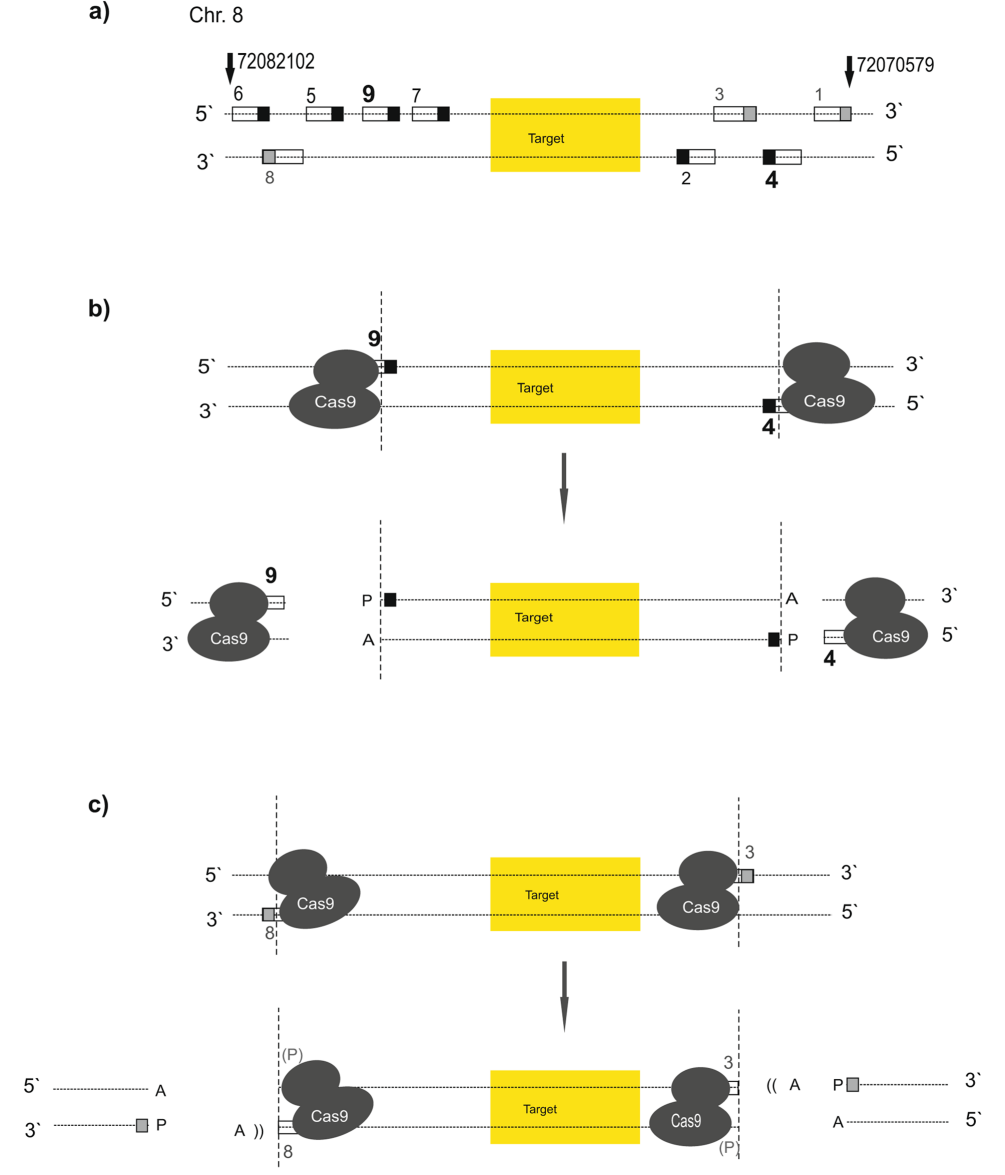
Positions of guide RNAs designed for the target, here the ***TRPA1*** promoter. Guide RNAs are numbered (compare Tables 1 and 3) and are depicted as boxes; PAM motifs are indicated by filled black squares, PAM motifs of “interfering” guide RNAs are indicated by filled grey squares. The guide RNAs yielding the highest number of calls per site (# 4 and # 9) are printed in bold. (a) This subfigure corresponds to Fig. 4 but is again shown in order to facilitate orientation in the next two subfigures. Position of guide RNAs relative to the target region on chromosome 8. Arrows mark outmost GRCh38 positions. (b) Correct position of guide RNAs enabling adapter ligation after cutting. (c) Blocking adapter binding by suboptimal positioned guide RNAs results in residual Cas9 molecules within the region, which has to be read, including the target

As already mentioned in the results part, it is of high importance to generate a sufficient number of calls per site in order to assess the accurate percentage values of methylation in a mixture of DNA strands. Although the usage of several guide RNAs upstream and downstream of the target region is recommended [15], surprisingly, a single pair of guide RNAs (# 4 and # 9) was shown to result in the highest number of calls per site obtained. The fact that we generated a lower number of calls per site during the establishment of the most suitable guide RNA strategy for our target, when we combined all guides, as opposed to using only guides 4 and 9 (Fig. 6a (corresponding to Fig. 4), Table 3), can be interpreted as follows: Usage of guide RNAs # 1, # 3, and # 8 probably hampers adapter binding, and thereby decreases the efficacy of the following sequencing reaction due to persisting Cas9 molecules within the region to be read. Despite a thermal Cas9-deactivating step in the protocol, the Cas9 enzyme, which is known to hardly dissociate from the DNA after cutting [21], remains bound to the DNA strand [15]. Since persistent Cas9 binding was shown to block DNA repair proteins from accessing Cas9-generated breaks [22], this observation might be also relevant for the experimental conditions in Cas9-mediated PCR-free enrichment protocols. It has already been described that the adapter binds preferentially on the 3’-side of a Cas9 cut, as the enzyme remains stably bound to the 5’-side of the sgRNA [15]. It is plausible that the first subsequent step after cutting, namely dA-tailing, may be hindered due to persisting Cas9 molecules since the polymerase requires at least 4 bp to bind to a DNA strand. Moreover, the DNA is single-stranded within the Cas9 molecule, and one of the strands is even hybridized with the guide RNA. Therefore, the prerequisites for polymerase binding are not fulfilled. Since y-shaped “Sequencing adapters are ligated primarily to Cas9 cut sides, which are both 3’ dA-tailed and 5’ phosphorylated” (manual “Cas9 targeted native barcoding”, Oxford Nanopore Technologies) [23], adapter ligation to DNA strands with bound Cas9 will be less effective. In addition, the phosphate group is also most likely inaccessible with persisting Cas9. All these factors make a reduced sequencing efficiency caused by persistent Cas9 molecules comprehensible. Since Cas9 binding occurs at the 5’-side of the guide RNA, and cutting close to the PAM region at the 3’-side, the blocking of adapter binding due to persisting Cas9 molecules after cutting is locatable in relation to the target (compare Fig. 6b and c). This should be considered when choosing sgRNAs for using the Oxford Nanopore Technologies “Cas-mediated PCR-free enrichment”-protocol. As depicted in Fig. 6b, the usage of sgRNAs # 4 and # 9 results in the accessibility of both ends for adapter ligation after cutting, which contrasts with the situation depicted in Fig. 6c. Therefore, the addition of the sgRNAs # 1, # 3, and # 8 leads to a reduced number of reads, since according to statistical probability, some DNA molecules will be blocked (at least unilaterally) for adapter ligation.

Despite the lower numbers of calls per site obtained by utilizing the “interfering” guide RNAs # 1, # 3 and # 8, methylation levels were measured within the same range for both guide RNA strategies (Table 3). Thus, nanopore data of the Crohn patient DNA with poor integrity can be assumed to be reliable, despite lower read numbers.

Although measurement of methylation levels is not 100% stable before reaching a number of calls per site of 100 (or a bit less) in our target CpGs, deviations are not high, even in the case of low read numbers. According to Oxford Nanopore Technologies a read depth of around 30 x is advised for assessment of methylation levels. Apart from the influence of DNA concentration and integrity, as well as the chosen guide RNAs, the condition of the flow cell and the corresponding number of active pores has an impact on read numbers. Thus, variations in the number of calls per site may occur regularly. Since one flow cell with a maximum load of five DNA samples can be used per nanopore run, it is not possible to circumvent the resulting divergence.

**To conclude**, according to the nanopore sequencing results, we obtained reproducible results with the chosen method of direct Sanger bisulfite sequencing in our previous studies [13, 14]. Methylation data obtained via nanopore sequencing are probably more accurate than those obtained via direct Sanger bisulfite sequencing (at least when reaching sufficient numbers of reads), due to the absence of bisulfite conversion and possible PCR amplification biases. However, the method of direct bisulfite sequencing is suitable for understanding the underlying regulatory interrelation when analyzing large cohorts. Furthermore, a higher reliability of data can be obtained by performing measurement in triplicates. Yet, other methods should be used for diagnostic purposes to allow valid statements for an individual. The advantages and disadvantages of the method of choice should be evaluated carefully before starting the analyses (Table 4). While direct Sanger bisulfite sequencing enables for a high throughput of samples (2 × 96 in one run, preparation time with amplification and sequencing PCRs 2 days) when analyzing a single promoter region, MinION nanopore sequencing is more labor- and time-intensive due to the maximum load of only up to five samples per flow cell (library preparation time 1 day). However, the utilization of sequencing devices, which can run several flow cells at once, is possible. Furthermore, several targets are analyzable in the same patient and run (multiplexing) and additionally pure sequence information like for example polymorphisms are obtained automatically in the same strand and can therefore be specifically correlated with the methylation status. Last but not least, one has to consider that high amounts of DNA (3 to 5 µg) are prerequisite for the target-based nanopore approach, whereas for Sanger bisulfite sequencing as little as 500 ng are sufficient.

**Table 4:** Overview of the special features of Sanger and Nanopore sequencing and their significance for practical work with both methods

	Sanger	MinION Nanopore
**Required DNA amount**	500 ng	3–5 µg
**Required DNA quality**	No special requirements	High Molecular Weight DNA
**Max. load of samples per run**	2 × 96	5
**Amount of readable targets per sample (multiplexing)**	1	Theoretically 20, but practically less, as the number of reads is crucial for the correct interpretation of the methylation data but is partly reduced by combining several sgRNAs.
**Reliability**	Depending on the target and maybe methylation level. A good quality of sequencing (as indicated by high base Quality Values) is prerequisite for interpretation of data.	Supposedly high. Sufficient numbers of calls per site are prerequisite for interpretation of methylation data.
**Costs**	Approximately 10,- €/sample If working in triplicates: 30,-€/sample	Approximately 150,- €/sample
**Preferably use for**:	-Large patient cohorts -One target of interest -Relative alterations of methylation status between groups	-Smaller number of samples -Multiplexing -Absolute methylation values of single/individual patients -Simultaneous gain of sequence information like polymorphisms in the same strand -To check Sanger sequencing strategies before cohort analysis

**To sum up**, Sanger sequencing could still be the method of choice when dealing with an investigation of large cohorts with an emphasis on relative differences of methylation rates between groups or time-points of a single target. Nanopore sequencing is more suitable when analyzing smaller study samples or even single individuals and a panel of several genes within each sample, rather than analyzing one single promoter region. However, methylation data obtained via direct Sanger bisulfite sequencing are adequate to draw conclusions in appropriately powered cohort studies. In case of large cohorts, it is recommendable to first countercheck the Sanger-strategy by nanopore sequencing in a few exemplary samples to make sure that Sanger sequencing will deliver reliable results in the targeted region of interest having its own individual conditions and requirements.

### Electronic supplementary material

Below is the link to the electronic supplementary material.

Supplementary Material 1

## Data Availability

Data are available from the corresponding author at Jahn.Kirsten@mh-hannover.de.
