# Supplementary material for: Comparison of methylation estimates obtained via MinION nanopore sequencing and sanger bisulfite sequencing in the TRPA1 promoter region
Source: BMC Med Genomics. 2023 Oct 23;16:257. doi: 10.1186/s12920-023-01694-6 (PMC10591399; doi:10.1186/s12920-023-01694-6)
Supplement: Supplementary file 1 — Supplementary Material 1 [file 12920_2023_1694_MOESM1_ESM.docx]

S1: supplemental table: Allocation of relative CpG positions in GRCh38.

| Original CpG Position | Corresponding position in GRCh38 |
| --- | --- |
| CpG -734 | 7.2076.349 |
| CpG -720 | 72.076.335 |
| CpG -628 | 72.076.243 |
| CpG -480 | 72.076.095 |
| CpG -452 | 72.076.067 |
| CpG -429 | 72.076.044 |
| CpG -412 | 72.076.027 |
